# A review of _urban water networks management using GIS

**DOI:** 10.1016/j.mex.2023.102261

**Published:** 2023-06-15

**Authors:** Kushal Patel, Seema Nihalani

**Affiliations:** a Parul Institute of Engineering and Technology, Parul University, Post Limda, Waghodia, Gujarat 391760, India; b Faculty of Civil Engineering, Parul Institute of Engineering and Technology, Parul university, Post Limda, Waghodia, Gujarat 391760, India

**Keywords:** GIS, Project, Construction management, Flood analysis, Water works, Spatial data, And non-spatial data, And spatial analysis, Hydraulic simulation, Pipe network optimization

## Abstract

Use of computer technology is common in the construction since the 90′s .Geographical Information System is a very useful technology in any data driven industry. The present paper reviews on the application and management of water works through GIS. GIS Data can be categorized as spatial and non-spatial data, which can be stored, manipulated, analysed and displayed with multiple users which can help in comprehensive solutions in a systematic way. GIS application is very common in construction industry, construction safety, flood studies, pipeline management which includes water works and sewerage. Project management through GIS is different from GIS based projects, the review briefs about the difference between the two. The pipe network management includes planning, designing, and management of the network, the planning is done using Remote sensing, photogrammetry drone or field survey method depending on the budget and objectives of the project. The designing of the network is done in GIS or separate application environment. The final step is the operations and management of the network which is in GIS.

## Review Highlights

• Use of GIS and its benefits.
• Very helpful in Planning and scheduling in the construction field.
• Major focuses on water works.

## Graphical abstract

**Figure:**
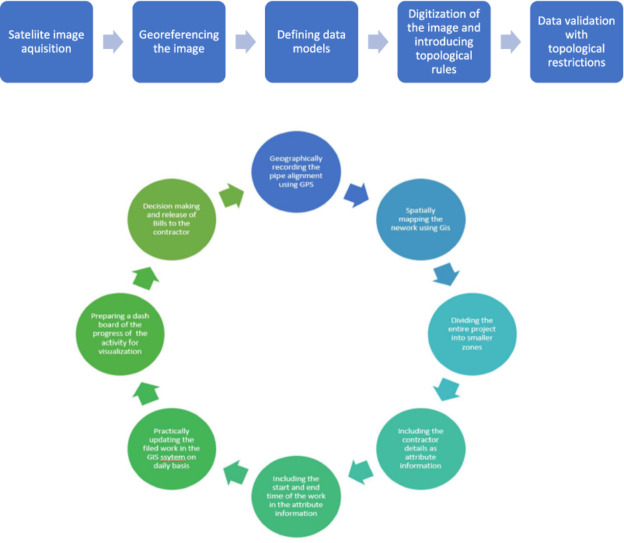

Specifications table

**Table:** 

Subject area:	Engineering
More specific subject area:	Civil Engineering
Name of the reviewed methodology:	GIS Application
Keywords:	*GIS, project, construction management, flood analysis, water works, spatial data, and non-spatial data, and spatial analysis, hydraulic simulation. Pipe network optimization*
Resource availability:	N.A

**Method details**

## Introduction to GIS and civil engineering

Use of computer technology is common in the construction since the 90′s. Geographical Information System is a very useful technology in any data driven industry. The present world's economy is driven by data analytics and information. Data visualisation, data storage and manipulation of data, development of applications are all the important features of GIS, [Technol. Manag 2001] which otherwise becomes complicated by using different applications for different purposes, example, in the construction industry, different applications like CPM for scheduling, 2D CAD for drawing. Water gems for designing, Etc.

GIS Data can be categorised as spatial and Non spatial data, which can be stored, manipulated, analysed and displayed with multiple users which can help in comprehensive solutions in a systematic way,

The GIS technology has basically 3 data models to represent the real world entities i.e. the point, line and polygon. These data are either collected from field survey/generated by using remote sensing satellite images by method of digitization [1]. This data can be represented on projection system with suitable geographical references. Each of the real world entities are stored in separate layers called shape files/Geodatabases in the form of point, line or polygon. The information related to these layers are stored in the attribute table of the shape file. The technology can conduct different analysis to obtain certain real world solution. These are called as spatial analysis. The inbuilt tools in the GIS application can modify different data into real world information.

## Applications of GIS in planning and construction and management

Application of GIS is wide in city/campus planning, flood modelling and construction management etc. Especially in city planning, campus planning and safety management. By adhering to 3D modelling of a city, it becomes easy to visualize the city/campus and understand the space constraints in the area. The 3D tools in the GIS system are used to simulate the functions of the buildings [1]. Apart from automation technology and Radar, GIS technology finds application in safety management. Construction activity management, water supply, electrical and sewage management etc.

Flood modelling is another important aspect where the runoff volumes are established using the rainfall data obtained from GPRS based telemetric rain gauges. The time series rainfall data is input into the hydraulic simulation model to obtain the flood hydrographs and the output is linked to GIS for analysis and visualisation purpose. GIS is very useful tool for flood hazard mapping, Flood zonation, Flood indexing and 3D analysis [2].

Several research have been done on safety management in construction industry. Sensor technology like GPS (Global positioning system), RFID, can give real time information which can predict accidents. Modelling technology like BIM, 3D, 4D, virtual reality are used for safety management [3]. GIS applied in construction safety management plays a key role in integrating the project activities and identification of accidental/emergency spots during the construction activities geographically. The GIS based navigation can be visualised in 3D to demonstrate the project planning, construction schedule and identify the accident zones and also the activities involving risks and accidents.

CPM method provides features of scheduling activities related to time basically splits the different activities based on start and end time. The schedule is basically associated with the drawings and its different components like the beam, column, walls, footing foundation etc. the actual start and end of the execution of these components is calculated in the CPM (Critical path method) say V.K Bansal

Following the design drawings and executing at the construction site leads to lot of gaps and improper monitoring of the whole activity. Several advancements in the technology has given way to 4D CAD, which is excellent in visualising the conflicts between scheduling, evaluate alternative construction methods. These methods though advanced, it is not very user friendly and customisable.

The application of the Gis technology in construction industry is in all crucial phases of the project i.e. during the feasibility study, planning design phase and also the construction Phase, shortlisting of the contractors, suppliers prequalification etc. [Mohamed N. Jeljeli, et al. 1993]. the site suitability study can be done using high resolution satellite imageries to decide on the site. The location of the site and the nearest path to reach the site, planning of the transportation of the materials, man power can be done using GIS technology. The estimation of the earth work and project costing can be done using the 3D tools in GIS. Especially for the high way alignment, utility network, rail network etc. It finds its application in the construction management where the progress monitoring, material and man power movement needs to be planned.

## GIS project management

GIS based project management is about managing computer application, implementation, client/stake holder, and also the project teams [Dr. Zakaria Yehia et al. 2021]. Project can be defined as a continuous tasks being performed to find solution/ outcome to a problem identified by the client/ stake holder. It includes defining a problem or a hypothesis, Planning and execution of several activities exclusively or parallel in a given period of time involving skilled manpower and technology. The results of project is tangible outcome to help the organisation or society.

**Fig. 1:**
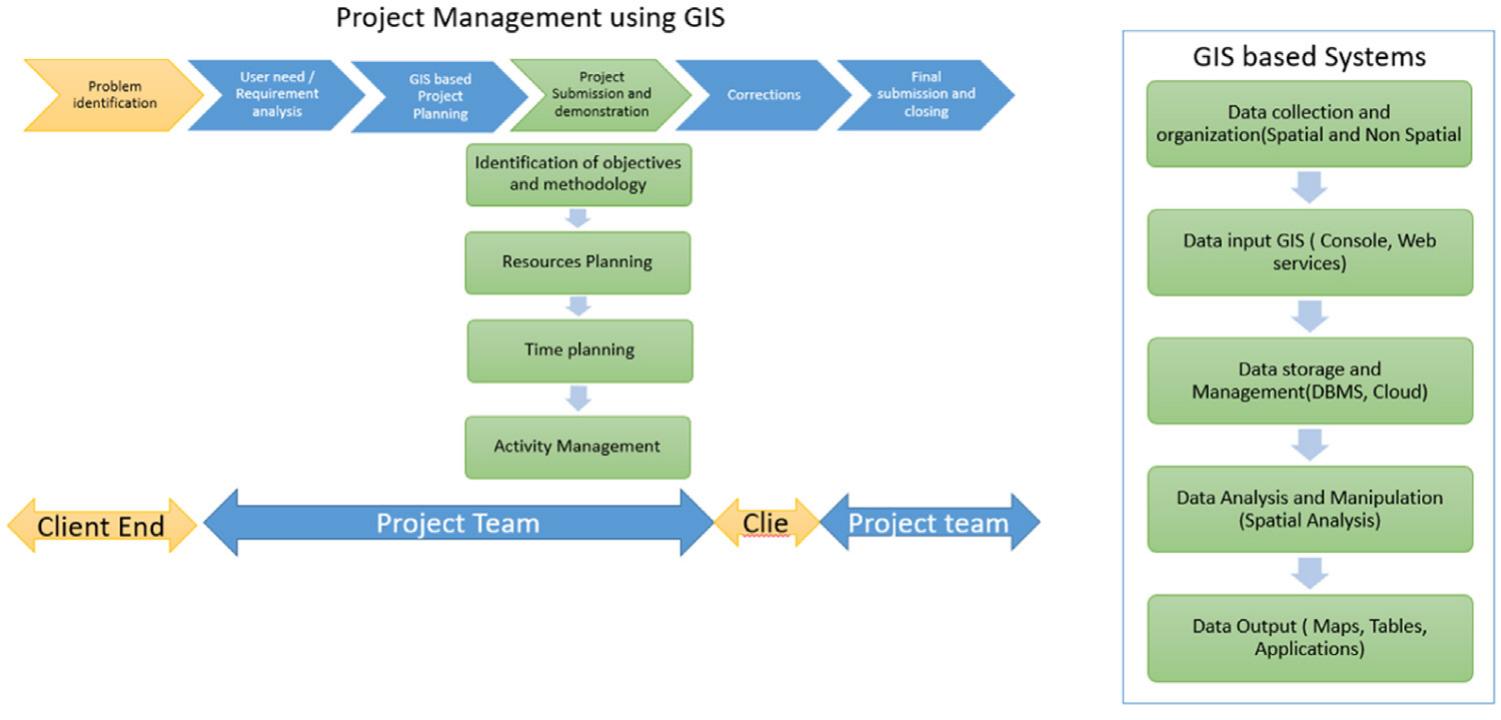
Components of GIS project.

**Fig. 2:**
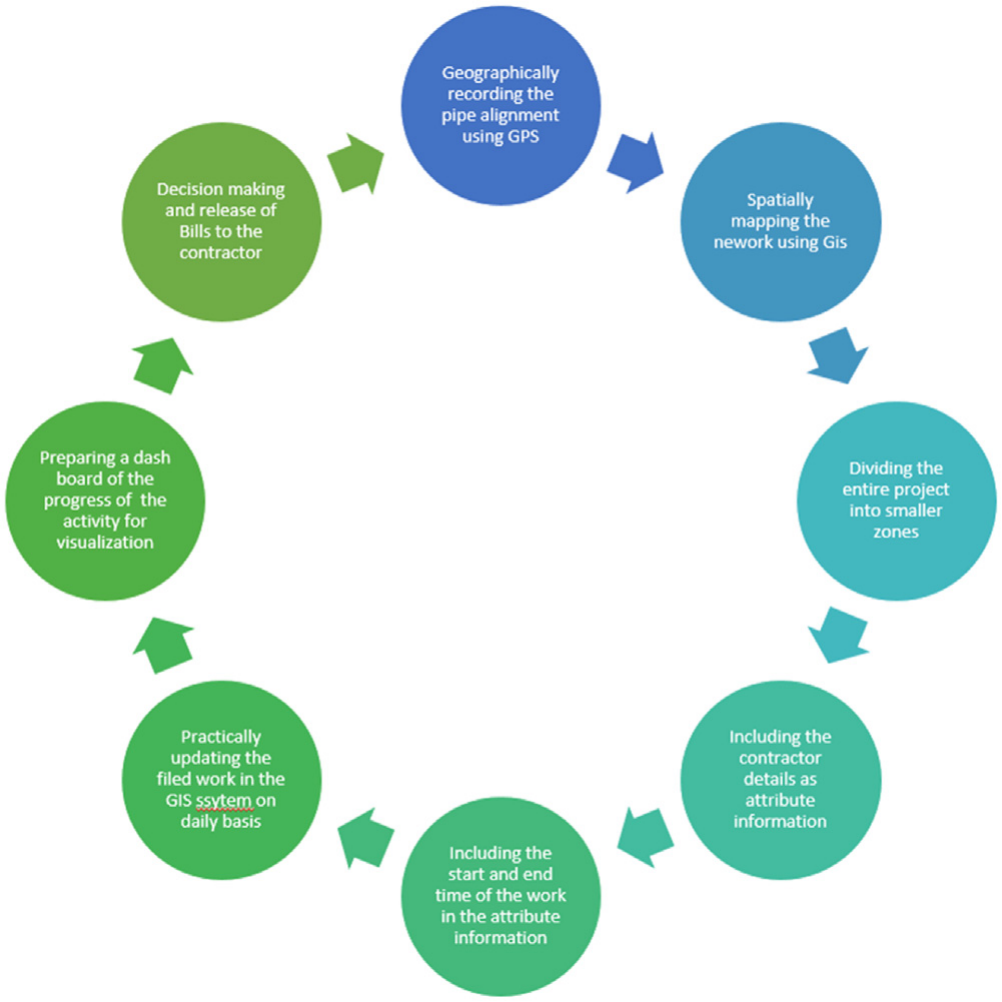
Project management using GIS technology.

When a project is initiated by an organisation it includes several departments like technical, HR, Quality etc. This is mainly to divide the roles and achieve maximum optimization of the project works. A Gis project will have the several components as shown in the Fig. 1 several projects can use GIS for Project management like monitoring and management of Utility services, Construction management etc. A GIS based project is different from project management using GIS. A GIS project is about using the technology in the defined project in order to achieve a solution or an outcome. A GIS based project management is about including all the schedule and activities of a project in a GIS system and using spatial tools to manage the project. Example: using GIS tool to monitor the progress of pipe laying work at a project site. The following process is involved in managing the project (Fig. 2).

The above process clearly explains how GIS can be used for managing a pipeline laying work process and helps in decision making. A successful project involves in complete understanding of the problem, and a good strategy to achieve the objectives of the project with required manpower and time.

## GIS for urban utility water management

The applications of GIS tools in urban water management is reviewed in this paper It is the most useful and upcoming applications of GIS based systems, The GIS based tools are widely used for Planning, operations and management of utility network.. Urban water networks includes both water supply and sewerage networks. Urban water networks management includes, the planning, designing, execution at the site and once pipe networks are layer, the operations and management of the whole network. The complexity in urban water management is; it involves large networks running across the municipal boundaries and different teams are working at different phases of the network. The usage of GIS shall provide a good visualization of the network and helps in the entire network management. The GIS tools are mainly used to plan the layout of the network, initially after the reconnaissance survey. The large network has to be laid on varying topography. Network layout can be planned using high resolution satellite images. Digital elevation model (DEM) is used to establish the alignment and calculate the earth work quantities using the 3D tools in GIS. Once the design of the alignment is completed the hydraulic design of the pipe network is carried out. Various computing models are used to design the network to arrive at the suitable pipe size based on the availability and distance of the water availability at the source. The modelled pipe network is then linked to GIS with all the design attributes of the pipe layout. The design parameters include the length, diameter, direction of flow, number of nodes, junctions, elevation etc. These are the critical information of water works which can be used for further operation and maintenance of the whole system during the laying and functioning of the water management system.

## GIS based network management system

### GIS based Pipe network data model

**Fig. 3:**
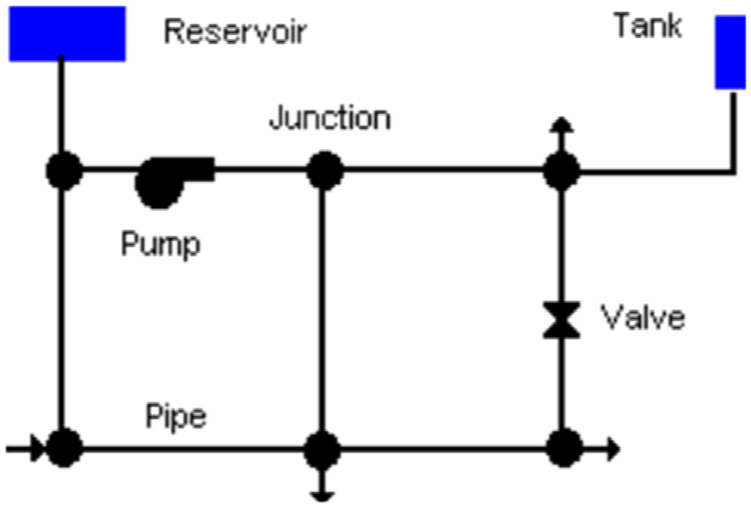
Elements of water supply network.

A typical pipe network system has the following feature entities (1) Reservoir (Source) (2) Pipe network (3) Pump system (4) Junction (5) Valve (6) Storage tank (Fig. 3). These feature entities have their specific attribute information which are very basic for pipe network modelling.

### Generation/creation of the Pipe network data

The network data is scientifically designed based on the water supply source available and the quantity of water to be distributed. The alignment of the network depends on topography of the area and the source to storage and supply distances. GIS helps in planning of the source and the distribution system. Then the alignment is planned using satellite image and the design entities are added during the design of the pipelines during the modelling of the network. The designing of a good data model involves in having the correct and the necessary information about the network. The data model should be finalized before the pipe network analysis [Pablo 2020]. During the simulation of the network the useful information is retained and any information which does add value to the decision making task is removed.

### GIS data creation and topological validation

**Fig. 4:**
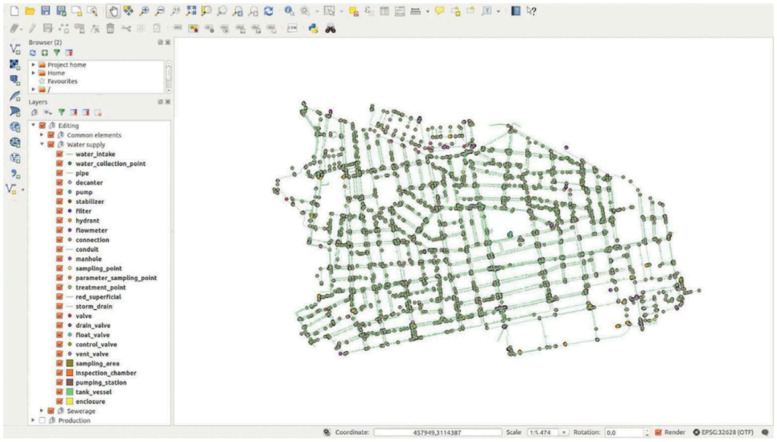
Map showing Sample pipe line network in GIS. *Source*: Abdulla Al-Rawabdeh GIS-based solution for urban water management GIS-based solution for urban water management.

**Fig. 5:**
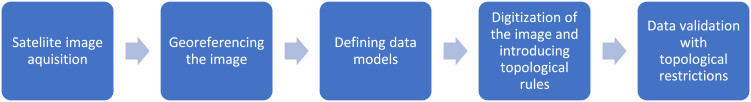
Process of data generation in GIS.

Pipe network data is created using GIS technology, either open source or proprietary software's. According to Pablo Fernández Moniz. All, GIS software's should include smart capabilities to ease the complex geoprocessing. The digitization procedure should have sufficient flexibilities to make useful changes. As data creation is one of the crucial steps in the pipe network management. All edits should happen during the design phase. Usually the edit database does all the changes and the production database shall not be disturbed. The production database gets updated frequently. The network design of a sample network is as shown in the Fig. 4. The process of pipe network data creation is shown below Fig. 5.

Topological validations are essential for pipe network modelling/ simulations. The input into the design model has to be clear of errors. This process begins after the editing of the network is completed with all additional and is ready to input into the simulation model. The validation of network data introduces robustness and integrity in the data. The process of validation involves adding some topological rules to the network data while digitisation.

**Fig. 6:**
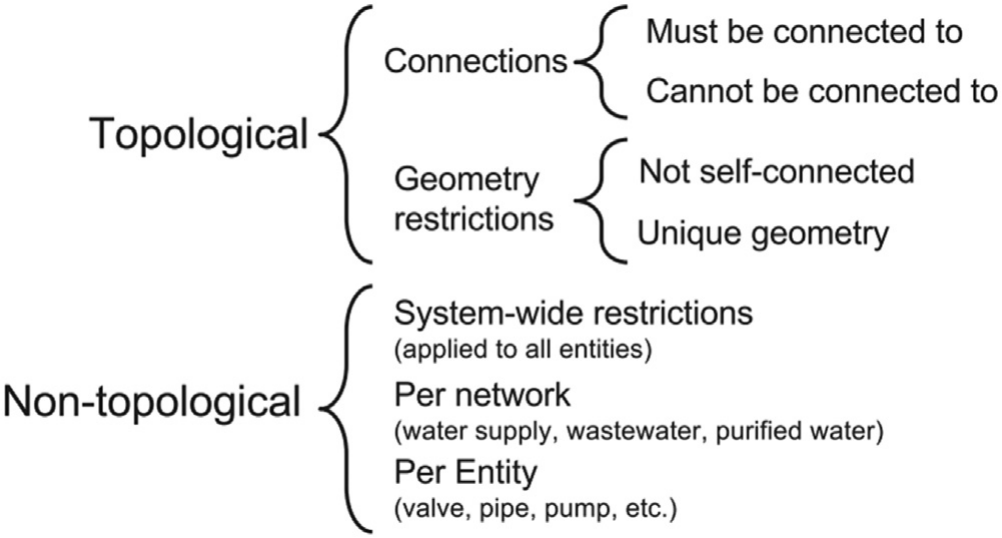
Restrictions and validations of pipe network data in GIS. *Source*: Water International A GIS-based solution for urban water management: Pablo Fernández Moniz.

According to Pablo Fernández Moniz et al. some of the simple topological validation for a network data includes (1) 2 lines must not overlap (2) pipe ends must always be connected (3) no two polygons must overlap etc. Once the data digitization is complete, the validation process in done by running through the restriction which are predefined in the data model. The restrictions can be Topological or non-topological. The details of restrictions for a pipe network data is as shown in Fig. 6 [4].

## Hydraulic modelling using GIS

**Fig. 7:**
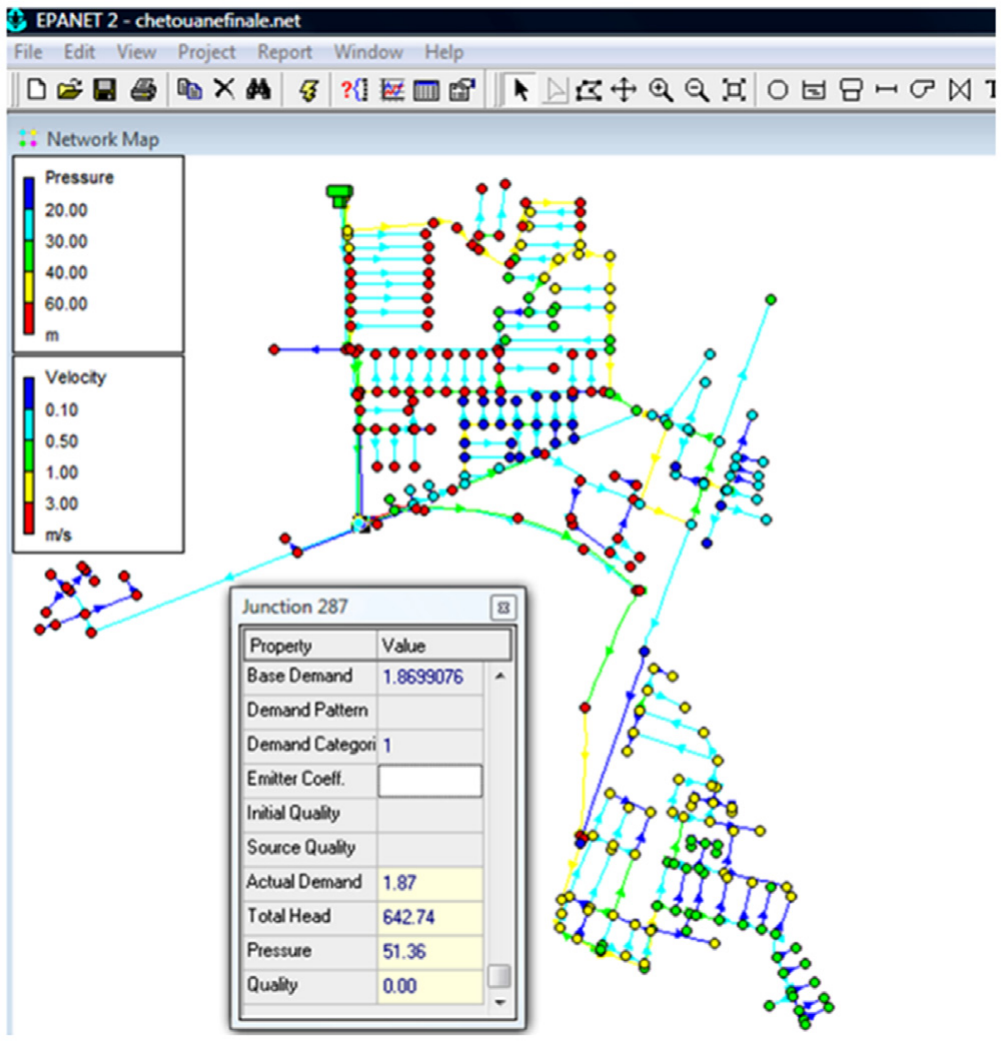
Pipe network information system of EPANET. *Source*: Cheŕifa Abdelbaki:Appl water Sci:Management of a water distribution network by coupling GIS and hydraulic modelling: a case study of Chetouane in Algeria.

Many models are available in the market for hydraulic modelling, it can be open source or proprietary software for eg: QGIS based on Post gre SQL Arc Gis using SQL etc. Pablo Fernández et al. 2020]. hydraulic modelling software's like EPA SWMM, EPANET, HEC RAS, HEC GeoRAS etc. can be integrated with GIS [5]. GIS software's help in storing the complex spatial data of the pipe networks such as pipe diameter, number of valves, number junctions, manholes, direction of water/sewerage flow etc. The development of the hydraulic modelling software have been improving to cope up with the complex issues pertaining to data quality, interoperability etc.[P*ablo* F*ernandez et al*. 2020]. The Role of the network simulation model is to analyse the network for its flow parameters like the pressure, velocity and direction. In a case study of Chetouane water distribution network in Algeria, EPANET was used for the hydraulic simulation where all the line networks in .DXF format were converted in the EPANET. Additional pipe network parameters like location of the tanks, pumps, valves nodes, junctions, manholes etc. were manually added using the features in the EPANET Fig. 7. Simulations were performed to arrive at consumption profile at each network. Pressure distribution, and the velocity distribution at the each of the networks. The advantage of such GIS based applications is, the Analysis and the visualisation happens in the same environment. Also back ground image data of the specific study area helps in understanding the site conditions in a scientific way. Also 3D simulations can be used to show the profile and the flow variations of the network.

## GIS integration with other applications

For effective pipe network management many drafting and design applications are coupled with GIS .for example CAD with GIS, EPANET with GIS, SWMM with GIS etc. [Cheŕifa Abdelbaki et al. 2016]. These applications can be used for identification of the best network routing, shortest path, optimised network alignment etc.

The CAD applications are used generate pipe network drawings, these files are in .DXF format and now these can be migrated to GIS environment in the form of shapefile format. Once the details of the network are in GIS, the attribute information is added to it. Checked for topology and the process follows as shown in Fig. 6.

In Applications like EPANET the details of network importing along with its characteristics can be done by the inbuilt operations within the EPANET [s (Ho et al. 2010)]. There are different layers of the pipe network which are stored differently in the form of point, line and polygon. Several spatial analysis and queries can performed in GIS. Basically to identify the best route, identify the network which are malfunctioning. to identify the age of the pipe network etc. [6].

## GIS based pipe alignment optimization

The present paper has discussed several applications and usage of GIS in water network management system. Another important and challenging application is optimization of a network. Automated optimisation is a better option than manual route optimization and GIS applications can handle this more easily [6], however for major pipelines, the best knowledge from the filed expert and stake holders can value add for decision making. The network Analyst tools in Arc GIS is the best option. Also Hierarchical Best Route algorithm which are model based algorithms gives best identification of the optimised routes. Which helps decision making says [6].

## GIS based operations and maintenance of the pipe networks

Once the pipe network is laid and fully functional with all connections between sources to distribution is completed by the execution contractor. The last and continuous process is operation and maintenance of the whole system. In reality the whole of this work is outsourced by the municipalities in urban areas to contractors who basically use technology to maintain their works.

GIS technology once again is a very useful tool which helps in this process. The main tasks of the maintenance team is to continuously monitor the networks. Usually the entire network right from source to distribution is divided into several zones and then handed over to different teams for the same. One of the well-known technology in the operations and maintenance of the pipe network is SCADA system (Supervisory control and Data acquisition). Which is a sensor based system this can be linked to GIS for monitoring of the network. The pipe network is connected with different types of sensors which constantly give information about the network parameters like flow, velocity, pressure and other variations. The SCADA system is linked to a GIS system, where all the details of the network can be visualised. The sensors constantly send information which can be visualised in the GIS interface, this will indicate the problematic variations in pressure and velocity. It also indicates the breakdown of pipes. All these information's are critical aspects of operation and maintenance. The SCADA technology thus saves time and energy to identify the defective lines.

## Conclusion

GIS based urban water works management has detailed about the concepts of GIS technology and use of this technology for project management for various applications of GIS, The process of GIS base project management highlights about the different steps in the project .the different sectors where GIS is widely used are construction management, flood studies, construction safety and water works management. The present paper widely reviews the GIS application in water works management. This mainly involves in planning, designing, construction and maintenance of the pipe networks. Remote sensing technology is used for planning and data generation of the pipe network. During the data generation three kinds of data model i.e. Line, Point and Polygon is used to create the network with suitable topology validations. Once the network is generated the design parameters of the network are obtained from RS and other hydraulic modelling software's like SWMM and EPANET. Hydraulic design of the network is either done in separate environment or linked to GIS. The paper also discuss about the optimised routing using algorithms which will result in efficient networks. Lastly the paper throws light on how the operations and maintenance of the network is done during the functioning of the network. Several research has been carried out on the hydraulic modelling and application integration in GIS. The future scope of the water works management is the quantification of water losses in the pipe network. It is very difficult to identify the leakages in the pipe network which is below the ground. Extensive research needs to be done in the pipe network management right from laying of the network to operation and management. It is very challenging task to determine the age of the pipe networks. As most of the networks are not mapped and no data on the pipe network is available. GIS plays a very important role in the mapping and database management of the network data. There needs to be lot of research done on the same.

## Ethics statements

Informed consent was obtained from participants or that participant data has been fully anonymized and the platform(s)’ data redistribution policies were complied with:

1) Authors declare to comply with the ethical guidelines of Journal.
2) All subjects gave their informed consent for inclusion before they participated in the study.

## CRediT authorship contribution statement

**Zhang San:** Conceptualization, Methodology, Software. **Priya Singh:** Validity tests, Data curation, Writing – Original draft preparation. **Wang Wu:** Visualization, Investigation. **Jan Jansen:** Supervision. **Ajay Kumar:** Software, Validation. **Sun Qi:** Writing – Reviewing and Editing. **Kushal Patel:** Conceptualization, Methodology, Software, Writing-Draft Preparation. **Seema Nihalani:** Validity test, Software, Supervision, Investigation, Validation, Reviewing.

## Declaration of Competing Interest

The authors declare that they have no known competing financial interests or personal relationships that could have appeared to influence the work reported in this paper.

## Data Availability

No data was used for the research described in the article.
